# A case–control study to assess the effectiveness of pertussis vaccination during pregnancy on newborns, Valencian community, Spain, 1 March 2015 to 29 February 2016

**DOI:** 10.2807/1560-7917.ES.2017.22.22.30545

**Published:** 2017-06-01

**Authors:** Juan Bellido-Blasco, Silvia Guiral-Rodrigo, Ana Míguez-Santiyán, Antonio Salazar-Cifre, Francisco González-Morán

**Affiliations:** 1 Epidemiology Department. Public Health Centre of Castelló (DGSP), Castelló, Spain; 2 Spanish Consortium for Research on Epidemiology and Public Health (CIBERESP), Instituto de Salud Carlos III, Madrid, Spain; 3 Universitat Jaume I (UJI), Castelló, Spain; 4 Surveillance and Epidemiologic Control Unit, Public Health Directorate, Regional Government of Valencia, Spain; 5 Epidemiology Department. Public Health Centre of València (DGSP), València, Spain

**Keywords:** Pertussis immunization, Matched case–control study, Pertussis and gestation, Pertussis newborn protection

## Abstract

In the Valencian Community (Spain), the programme of maternal pertussis vaccination during pregnancy started in January 2015. The objective of this study was to estimate in this region the vaccine effectiveness (VE) in protecting newborns against laboratory-confirmed pertussis infection. A matched case–control study was undertaken in the period between 1 March 2015 and 29 February 2016. Twenty-two cases and 66 controls (+/− 15 days of age difference) were included in the study. Cases were non-vaccinated infants < 3 months of age at disease onset testing positive for pertussis by real-time PCR. For every case three unvaccinated controls were selected. Odds ratios (OR) were calculated by multiple conditional logistic regression for association between maternal vaccination and infant pertussis. Other children in the household, as well as mother- and environmental covariates were taken into account. The VE was calculated as 1 − OR. Mothers of five cases (23%) and of 41 controls (62%) were vaccinated during pregnancy. The adjusted VE was 90.9% (95% confidence interval (CI): 56.6 to 98.1). The only covariate in the final model was breastfeeding (protective effect). Our study provides evidence in favour of pertussis vaccination programmes for pregnant women in order to prevent whooping cough in infants aged less than 3 months.

## Introduction

Pertussis persists as an infection of global public health importance. Many countries with long-standing vaccination programmes have reported a resurgence of pertussis, despite sustained high vaccine coverage [1-4].

In October 2012, the United States and United Kingdom became the first countries recommending that pertussis-containing vaccine (tetanus, diphtheria, acellular pertussis (Tdap)) should be routinely offered to women in every pregnancy [5]. Tdap immunisation during gestation is thought to augment the transplacental transfer of pertussis-specific IgG [6]. This process may be affected by multiple factors including placental integrity, total IgG concentration in maternal blood, time of immunisation, and time elapsed between immunisation and delivery.

Although there is no generally accepted level of pertussis-specific antibodies that would confer protection against infection [7], results reported from some countries since 2012 [8], on maternal pertussis immunisation at any time before or after pregnancy improving protection of very young children are encouraging. On the other hand, we do not have a correlate for protection for all vaccines, but can still demonstrate that they offer protection in field studies.

Since January 2015, the Valencian Community’s General Directorate of Public Health has recommended that pregnant women be offered a single dose of Tdap vaccine between 27 and 36 weeks of gestation, as a measure to temporarily protect infants in a period following birth and before these infants receive vaccination according to the schedule.

The main objective of this study was to estimate, in our region, the pertussis vaccine effectiveness (VE), when given to pregnant women, in protecting newborns against laboratory-confirmed pertussis infection using a case–control study design.

## Methods

### Setting and study

Whooping cough is a notifiable disease in Spain. Notified cases do not necessarily have to be PCR laboratory-confirmed, but confirmation by this method frequent. The current recommended infant schedule is: one dose of vaccine at 2 months-old, a second at 4 months-old, a third at 6 months-old, and a fourth at 18 months-old, with a final dose between the age of 5 and 9 years. 

A prospective matched case–control study was carried out through one year in a dynamic population. The study covered the whole territory of the Valencian Community (5 million inhabitants). 

### Participants

All unvaccinated pertussis infants notified in the Valencian Community during the study period had been PCR-laboratory-confirmed. Cases were defined as unvaccinated infants less than 3 months-old, with pertussis microbiological confirmation by PCR. They were identified from a computerised mandatory notification system (AVE, Análisis de Vigilancia Epidemiológica) from 1 March 2015 until 29 February 2016. 

For every case three paired controls by age, with an age difference of less than 15 days, were included. Two of these three controls were infants who had consulted the same paediatrician/family doctor practice as the case, and had presented to this practice either for a routine assessment or for a consultation due to ill-health. In order to avoid a possible overmatching in this setting, we selected a third control fulfilling the same criteria as the prior described controls, but from the maternity clinic where the case was born. Like the cases, controls were unvaccinated. Absence of whooping cough in controls was confirmed by checking clinical records and by phone interviews with parents and paediatricians/family doctors. The children with any previous episodes of cough and bronchiolitis were excluded. 

### Sample size

Taking as reference, 17% vaccination of the mothers among the cases [9], with a vaccine effectiveness of 90% and a statistical power of 80%, the number of children needed for the study was 52 (13 cases and 39 controls).

### Information on participants

Information from cases was obtained from paediatricians and parents either by face-to-face interviews during the period of their hospitalisation, or by phone, for cases who were not hospitalised, to avoid misclassification. Information from matched controls was collected less than 5 days after case notification by trained nurses. A questionnaire elaborated specifically for the study was used to collect medical information and exposure risks from child, mother and environment in both groups.

Presence or absence of disease in the newborn and vaccination of the mother during the pregnancy were the main variables. The vaccination status of all mothers in the study was verified in the Register of Vaccinations of the Valencian Community; we collected vaccination dates. Using the same register, it was also checked that none of the cases and controls were vaccinated.

### Case–control study

Risk covariates were classified in three groups: (i) Children covariates: date of birth, age in days, sex, city of residence, birth weight, Apgar test, breastfeeding; (ii) Mothers’ covariates: age, pregnancy week of the childbirth, precedent of whooping cough disease during the 10 previous years, precedent of whooping cough vaccine during the 10 previous years, immigrant background, level of education (low: elementary school; middle: secondary school; high: university) and employment status (employed vs unemployed); (iii) Environmental covariates: number and age of relatives in the household, number of them at school, smoking habits of the parents at home.

Simple and adjusted odds ratios (OR) were calculated by means of logistic conditional regression. Simple OR were first calculated. Variables potentially associated with pertussis in the newborn (i.e. with a p value < 0.10) were subsequently entered in a stepwise multivariate model, in which the variable with lowest p value at each step was removed, to produce a final model. 

The vaccine effectiveness (VE) was calculated as 1 − OR. Estimations and 95% confidence intervals were obtained using the STATA version 12 package.

To investigate how the VE varies depending on the setting from where controls were recruited, the VE was also calculated (i) with cases and controls paired by paediatrician/family doctor practice, or (ii) with cases and controls paired by maternity ward/clinic. Cases are the same in each sub-analysis, but their matched controls either originated only from the paediatric/family doctor practice where cases presented for treatment, or only from the maternity clinic where cases were born. When sample size was limited, exact methods of logistic regression stratifying for number of pair were applied.

In order to examine with more detail a possible interaction effect between breastfeeding and vaccination, a stratified analysis was carried out.

### Ethical issues

Informed consent was obtained from all participants before the interview. The principal researcher consulted with the Ethics Committee of the Health Department of the Valencian Community, which approved the study.

## Results

All cases took part in our study. One control from the maternity group did not participate and was replaced with another one chosen among infants who had consulted at same paediatrician/family doctor practice as the case it was paired with. Moreover, during the process of identifying controls, between two and three control infants per case were excluded on the basis of recent cough/bronchiolitis according to clinical records. However, subsequent to these exclusions, we could still interview three controls for each case (66 controls). 

### Characteristics of participants

**Figure 1:**
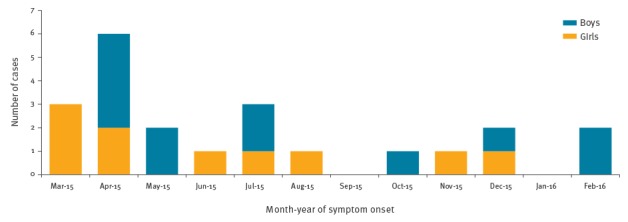
Cases of pertussis among newborns, by month of symptom onset, Valencian Community, Spain, 1 March 2015–29 February 2016 (n = 22)

Overall a total of 22 cases were identified, most of them during the first half of the study period (Figure 1).

Of 22 cases, 18 were hospitalised. The mean of age of cases was 46 days (range 10 to 82 days). The demographic characteristics and the OR estimated for variables hypothesised to be associated with pertussis are shown in Table 1.

**Table 1:** Characteristics of the participants^a^ in the case–control study to assess the effectiveness of pertussis vaccination during pregnancy on newborns, and univariate analysis results, Valencian Community, Spain, 1 March 2015–29 February 2016 (n = 88 participants)

Characteristic		Cases (n = 22)	Controls (n = 66)	OR simple (95% CI)	P value^b^
Mother vaccinated		5	41	0.080 (0.017 to 0.371)	0.001
Sex (girls)		10	29	0.932 (0.331 to 2.62)	0.895
Birthweight mean (g)		3,291	3,180	1.001 (0.999 to 1.002)	0.226
Birthweight <2,500 g		2	1	0.166 (0.015 to 1.83)	0.143
Gestation weeks at birth (mean)		38.4	38.7	0.868 (0.634 to 1.19)	0.378
Apgar <10 (percentage)		10	23	1.69 (0.576 to 4.94)	0.339
Feeding	Infant formula	11	17	1	NA
	Mixed feeding	4	9	0.646 (0.158 to 2.64)	0.543
	Breastfeeding	7	40	0.227 (0.066 to 0.775)	0.018
Breastfeeding (yes/no)^c^		7/15	40/26	0.259 (0.081 to 0.832)	0.023
Foreign mother		3	9	1.00 (0.202 to 4.95)	1.000
Mother's age: mean (years)		32.6	33.4	0.968 (0.878 to 1.07)	0.521
Educational level^d^		14	24	3.04 (1.10 to 8.43)	0.033
Mother's position^e^		8	27	0.834 (0.714 to 2.19)	0.714
Mean number of cohabitants in the participant’s household		3.14	2.73	1.45 (0.914 to 2.31)	0.114
Mean number of adults (>14 years-old) cohabiting in the participant’s household		2.14	2.08	1.17 (0.518 to 2.66)	0.701
Mean number of 10–14 year-olds cohabiting in the participant’s household		0.18	0.14	1.32 (0.408 to 4.28)	0.641
Mean number of 5–9 year-olds cohabiting in the participant’s household		0.32	0.21	1.41 (0.608 to 3.26)	0.424
Mean number of 0–4 year-olds cohabiting in the participant’s household		0.50	0.26	2.76 (0.994 to 7.67)	0.051
Schoolchildren of 3–11 years-old cohabiting with the participant in the participant’s household		16	32	4.34 (1.13 to 16.6)	0.032
Habit of smoking at home		3	8	1.14 (0.276 to 4.75)	0.853

CI: confidence interval; NA: not applicable.

^a^ Participants included newborns unvaccinated for pertussis, who were less than 3 months-old.

^b^ Comparison was done by conditional logistics regression.

^c^ Yes: exclusively breastfeeding; No: mixed feeding (formula and breastfeeding) or formula feeding.

^d^ Reference for equation: middle–low.

^e^ Reference for equation: unemployed mother.

Mothers of five cases compared to mothers of 41 controls were vaccinated. All vaccinated women had their vaccine administered between weeks 28 and 36 of gestation, and 15 to 89 days before childbirth. The proportion of vaccinated mothers increased during the study period from 24 of 59 in the first half of the study to 22 of 29 at the end (p value = 0.003). No mother had been vaccinated or affected by whooping cough during the previous 10 years. Among highly educated mothers 31 of 50 were vaccinated; among low, 15 of 38 (p value = 0.030). Among highly educated mothers, 30 of 50 were breastfeeding; among low, 17 of 38 (p value = 0.197).

The simple OR of vaccination in pregnancy was 0.080 (95% confidence interval (CI): 0.017 to 0.371). Other variables with statistically significant association were: breastfeeding, level of education and presence of children under 15 years-old in the home.

Adjusting by these variables at the beginning (Model 1, Table 2) and eliminating those which lost statistical significance, only vaccination and breastfeeding remained related with the protection against whooping cough (Model 3, Table 2). The adjusted VE did not change substantially, being 90.9% (95% CI: 56.6 to 98.1) for the final model. The interaction between vaccination and breastfeeding in the model with both variables was not significant (p value = 0.132). The replacement of the variable breastfeeding by a dummy variable with three categories did not modify the results.

**Table 2:** Result of successive multivariate analysis of potential factors associated with pertussis in newborns, Valencian Community, Spain, 1 March 2015–29 February 2016 (n = 88)

Characteristic	Model 1^a^		Model 2^a^		Model 3^a^	
	Adjusted OR (95% CI)	P value	Adjusted OR (95% CI)	P value	Adjusted OR (95% CI)	P value
Mother vaccinated	0.127 (0.025 to 0.658)	0.014	0.116 (0.024 to 0.567)	0.007	0.091 (0.019 to 0.434)	0.003
Breastfeeding	0.365 (0.095 to 1.40)	0.141	0.350 (0.092 to 1.32)	0.121	0.301 (0.079 to 1.15)	0.080
Schoolchildren in the household^b^	2.17 (0.397 to 11.9)	0.370	2.44 (0.484 to 12.3)	0.280	NA^a^	NA^a^
Educational level^c^	1.33 (0.347 to 5.13)	0.675	NA^a^	NA^a^	NA^a^	NA^a^

NA: not applicable.

^a^ The variable with the highest p value in each consecutive model (1, 2, etc.), was removed in the next model.

^b^ Schoolchildren include children aged 3 to 11 years.

^c^ Reference for equation: middle–low.

**Figure 2:**
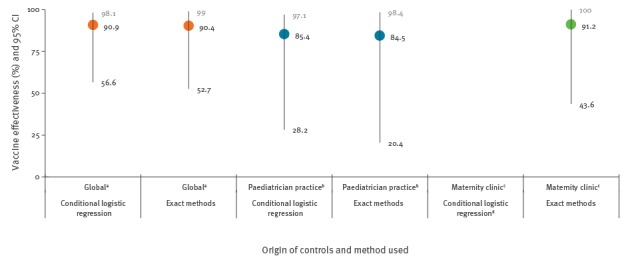
Vaccine effectiveness and 95% confidence interval in function of the origin of the controls, Valencian Community, Spain, 1 March 2015–29 February 2016 (n = 88)

VE: vaccine effectiveness; CI: confidence interval.

^a^ Global: controls either respectively consulted at the same paediatrician/family doctor practice as the cases, or were born at the same respective maternity clinic.

^b^ Paediatrician practice: controls consulted the same the paediatrician/family doctor practices where the respective cases presented.

^c^ Maternity clinic: controls were born at the same maternity clinic as the respective cases.

^d^ The conditional model in the maternity subgroup did not converge, due to small sample size, and does not give odds ratios.

Results obtained from the sub-studies with controls who only originated from the same paediatrician/family doctor practice as the cases, or with controls only coming from the maternity clinics where the cases were born, also showed a protective effect of vaccination but with small differences between them. The conditional model in the maternity subgroup did not converge, due to small sample size, and does not give ORs (Figure 2).

In spite of the fact that the interaction between vaccine and type of feeding in the whole sample was not statistically significant, we carried out an analysis taking as reference newborns with non-vaccinated mothers and artificial feeding, excluding newborns with mixed feeding (13 children) as shown in Table 3. Mother vaccination during pregnancy has a VE of 95.4% and the VE improves slightly with breastfeeding, i.e. to 96.7%.

**Table 3:** Assessment of vaccine effectiveness in function of breastfeeding or artificial feeding by means of conditional logistic regression model, Valencian Community, Spain, 1 March 2015–29 February 2016 (n = 75)

Vaccine status of mother and type of feeding	Cases (n = 18)	Controls (n = 57)	OR (95% CI)	P value	Effectiveness (95% CI)
Non-vaccinated and artificial feeding	9	6	1	Ref	Ref
Non-vaccinated and breastfeeding	4	15	0.166 (0.017 to 1.65)	0.126	83.4% (-65 to 98.3)
Vaccinated and artificial feeding	2	11	0.046 (0.003 to 0.639)	0.022	95.4% (36.1 to 99.7)
Vaccinated and breastfeeding	3	25	0.033 (0.003 to 0.361)	0.005	96.7% (63.9 to 99.7)

CI: confidence interval; OR: odds ratio; ref: reference.

Newborns with mixed feeding excluded.

Finally we observed a protective effect of the breastfeeding among children from non-vaccinated mothers, i.e. a VE of 83.4%, but with wide confidence intervals.

## Discussion

Two aspects stand out in this study: First and more importantly, we have observed a high effectiveness of the pertussis vaccine. Around 90% of the cases in newborns under 3 months-old might be avoided by vaccinating their mother in the third trimester of pregnancy. Second, the results also suggest a possible protective effect of breastfeeding in the absence of vaccination.

The magnitude of the VE in this study is in agreement with two previous studies, which report VEs of 91% [10] and 93% [9]. Armirthalingam et al. [10] found a VE of only 38% when they restricted their analysis to vaccinated mothers 0–6 days before childbirth or 1–13 days later. In our study all the mothers were vaccinated at least 2 weeks before the childbirth.

We had complete information on all the cases selected for the study and their paired controls, which allowed us to analyse by conditional logistic regression. Our results showed a strong protective effect of maternal vaccination once adjusted for type of feeding of the newborn, without observing a degree of substantial confounding from other variables. The estimations of the VE obtained with conventional methods of unconditional logistic regression were slightly lower (data not shown).

We agree with Dabrera et al. [9] that the estimated effectiveness could be a combination of direct biological effect, produced by the antibodies that the mother transfers to her child, with the indirect protection due to the reduction of the risk of domiciliary transmission from the mother who is protected against whooping cough. The possible protective effect of breastfeeding may originate from natural components of breast milk or specific anti-PT IgA produced by the mother as a result of vaccination, since concentration is high in colostrum and lasts at least until the eighth week post-partum [11].

We acknowledge that there are limitations of our observational study, since the comparability of the groups could be compromised. The mothers who choose to be vaccinated can present features different from those who do not do it [12]. In fact, the women who got vaccinated during pregnancy, tended to also follow the vaccination schedule more thoroughly for their previous children too. This could introduce a protection bias following the effect of maternal vaccination. In order to control for confounding, several multivariate sequential analyses were carried out. According to the study of Quinn et al. [13], exposure to cohabiting school children and level of educational attainment of mothers were associated with whooping cough in infants. In our study, this was only observed in the simple analysis, but not in the multivariate one. There could be other confounders that we have not evaluated. Among them could be the maternal antibody level at the beginning of pregnancy [14], or some genetic polymorphisms linked with vitamin D [15].

With regard to the eventual modification of effect influenced by the type of feeding, it would be necessary to have a sufficient number of children in every stratum to analyse this aspect with more precision. Our results suggests that breastfeeding should be a factor to be considered in the future, in other studies with a larger sample size and this starting hypothesis.

In this study, all unvaccinated cases less than 3 months-old, who were notified to the AVE, were included, generally covering the whole autonomous community. We cannot exclude some bias in case ascertainment, because milder cases are frequently missed by healthcare systems. In our study, 18 of the 22 reported cases were hospitalised, reflecting the high proportion of infants diagnosed with pertussis who are treated at the hospital. The response rate in our study was 100%, so we believe that there is no risk of bias of selection by non-response.

An interesting aspect is that, a progressive decrease of the incidence of cases in children less than 3 months-old was observed (16 of 22 cases in the first half of the year) along the study period. This could be consequence of the gradual vaccination programme implementation in pregnant women during the period, supporting the hypothesis of its effectiveness. But in the absence of data from other age groups, this evolution cannot be directly attributed to vaccination. Also, the duration of the study, one year, does not allow to rule out a seasonal effect.

We think that the robustness of the study rests on the quality of the information from principal variables. All cases were confirmed by clinical microbiological tests. Recent medical records were reviewed for controls avoiding children with whooping cough symptoms, among those not diagnosed. For both mothers of cases and controls vaccination status was verified and the dates of vaccine administration were obtained.

In spite of existing limitations, we believe that our findings offer results with sufficient internal validity, the results agree with other published papers and have biological plausibility. We have observed, while reducing several of the possible biases, a robust association between vaccination during pregnancy and whooping cough.

Our results, from an external validity perspective, could be implemented for pertussis prevention in infants less than 3 months-old. We have neither investigated effectiveness on a middle or long-term in older children, nor possible interference of the mother’s vaccination when children will be vaccinated with three doses during the first year of life (2, 4 and 6 months-old) [16].

Finally, at a time in which whooping cough presents new epidemiological features and new challenges for its control [17], our study, together with others recently published in other contexts [18-20], provide enough evidence in favour of the implementation of vaccination programmes for pregnant women in order to prevent whooping cough in infants.
